# Novel *LHX8* variants associated with distinctive oocyte morphological abnormalities and maturation arrest in primary infertility

**DOI:** 10.1186/s13048-026-01978-2

**Published:** 2026-01-21

**Authors:** Yusuke Sako, Hidehito Inagaki, Akira Yanagihara, Koichi Kinoshita, Kaname Nakayama, Yasuyuki Mio, Keitaro Yumoto, Rei Hirata, Toshihiro Habara, Haruki Nishizawa, Hiroki Kurahashi

**Affiliations:** 1 Division of Molecular Genetics, Fujita Health University, 1-98 Dengakugakubo, Kutsukake- cho, Toyoake, Aichi 470-1192 Japan; 2 Department of Obstetrics and Gynecology, Fujita Health University, 1-98 Dengakugakubo, Kutsukake-cho, Toyoake, Aichi 470-1192 Japan; 3 Kyoto IVF Clinic, Teianmaenocho 613, Teramachi-dori Shijo-sagaru, Shimogyo-ku, Kyoto, 600-8031 Japan; 4 Mio Fertility Clinic, 2-1-1 Kuzumo-Minami, Yonago, Tottori 683-0008 Japan; 5 Okayama Couple’s Clinic, 285-1 Tsudaka, Kita-ku, Okayama, Okayama 701-1152 Japan

**Keywords:** LHX8, Oocyte degeneration, Maturation arrest, Primary infertility, Oocyte/Zygote/Embryo maturation arrest (OZEMA)

## Abstract

**Background:**

*LHX8* gene encodes a germ cell specific transcription factor that is required for oocyte development. We evaluated two unrelated women with primary infertility who showed reproducible oocyte abnormalities across in vitro fertilization cycles, and we performed genomic and functional assays to clarify the role of *LHX8*.

**Results:**

Whole exome sequencing identified heterozygous loss-of-function variants in *LHX8* (NM_001001933.1) in both patients: c.778 C > T (p.Gln260Ter) in family 1 and c.581-1G > A in family 2. Both variants met the American College of Medical Genetics and Genomics criteria for likely pathogenicity. The two patients had high proportions of degenerated or immature oocytes and showed consistent morphologic features, including multiple cytoplasmic vacuoles, impaired zona pellucida function with accumulation of sperm in the perivitelline space, and poor embryo development. The splice site variant was inherited from a fertile mother, which indicates incomplete penetrance. A minigene assay confirmed the use of a cryptic acceptor site that produced a one nucleotide deletion and a frameshift, consistent with loss of function.

**Conclusions:**

These findings expand the phenotypic spectrum of *LHX8* related infertility and provide mechanistic evidence that partial reduction of *LHX8* activity compromises oocyte quality. Recognition of the characteristic morphology may guide genetic testing and counseling in cases of unexplained infertility.

**Supplementary Information:**

The online version contains supplementary material available at 10.1186/s13048-026-01978-2.

## Background

Infertility is a global health issue that will likely continue to rise through 2040. In 2021, it affected an estimated 110 million women and 55 million men worldwide, with a nearly two-fold higher prevalence in women than in men [1]. Within female infertility, oocyte maturation abnormalities (OMAS) and oocyte, zygote, or embryo maturation arrest (OZEMA) are increasingly recognized as important causes. Assisted reproductive technology (ART) makes it possible to observe oogenesis and early embryogenesis in detail and has refined these concepts. OMAS covers a spectrum of oocyte maturation defects, including degenerated or dysmorphic oocytes, empty follicle syndrome, oocyte maturation arrest (OMA), resistant ovary syndrome, and maturation disorders associated with primary ovarian insufficiency (POI) [2]. OZEMA refers to developmental arrest at the oocyte, zygote, or early embryo stage [3]. Successful oocyte maturation requires precise temporal and spatial control of gene networks that regulate meiotic progression, cytoskeletal dynamics, and organelle distribution. Disruption of these programs results in maturation failure and infertility. Genetic factors that influence these processes have attracted growing attention, and several infertility related genes have been identified, including *TUBB8* and *ZP1* [4]. *TUBB8* variants cause oocyte maturation arrest through microtubule disruption, while *ZP1* variants cause fertilization failure through defects in the zona pellucida. These insights have advanced our understanding of human gametogenesis and stimulated new approaches to diagnosis and counseling.

*LHX8* belongs to the LIM homeobox family and has a central role in embryonic development through transcriptional regulation of pattern formation and cell fate [5]. LIM homeodomain proteins contain two N terminal LIM domains that mediate protein interactions and a C terminal homeodomain that binds DNA in a sequence specific manner [6]. In mammals, *LHX8* is expressed mainly in the ovary and functions as a germ cell specific transcription factor that is essential for oocyte differentiation and survival. In mouse models, deletion of *Lhx8* leads to rapid oocyte loss and impaired follicle development from the primordial to the growing stages [7]. The human *LHX8* gene on chromosome 1p31.1 has ten exons and is highly conserved across mammals, which indicates essential reproductive functions.

Loss of function variants in *LHX8* have recently been reported as a cause of oocyte maturation arrest and female infertility [8]. Although homozygous loss in mice causes severe reproductive abnormalities, heterozygous *LHX8* variants may be sufficient to cause infertility in humans, which suggests species specific differences in gene dosage sensitivity. Reported cases show diverse phenotypes, including arrest at the germinal vesicle or metaphase I stage and morphological abnormalities in retrieved oocytes [8]. However, detailed morphological characterization in human heterozygous *LHX8* variants remains limited.

Here we report two women with primary infertility who carry novel *LHX8* variants. Both showed distinctive oocyte morphology and developmental arrest. Our findings expand the phenotypic spectrum and provide new insight into the function of *LHX8* in human reproduction.

## Methods

### Clinical samples

**Fig. 1:**
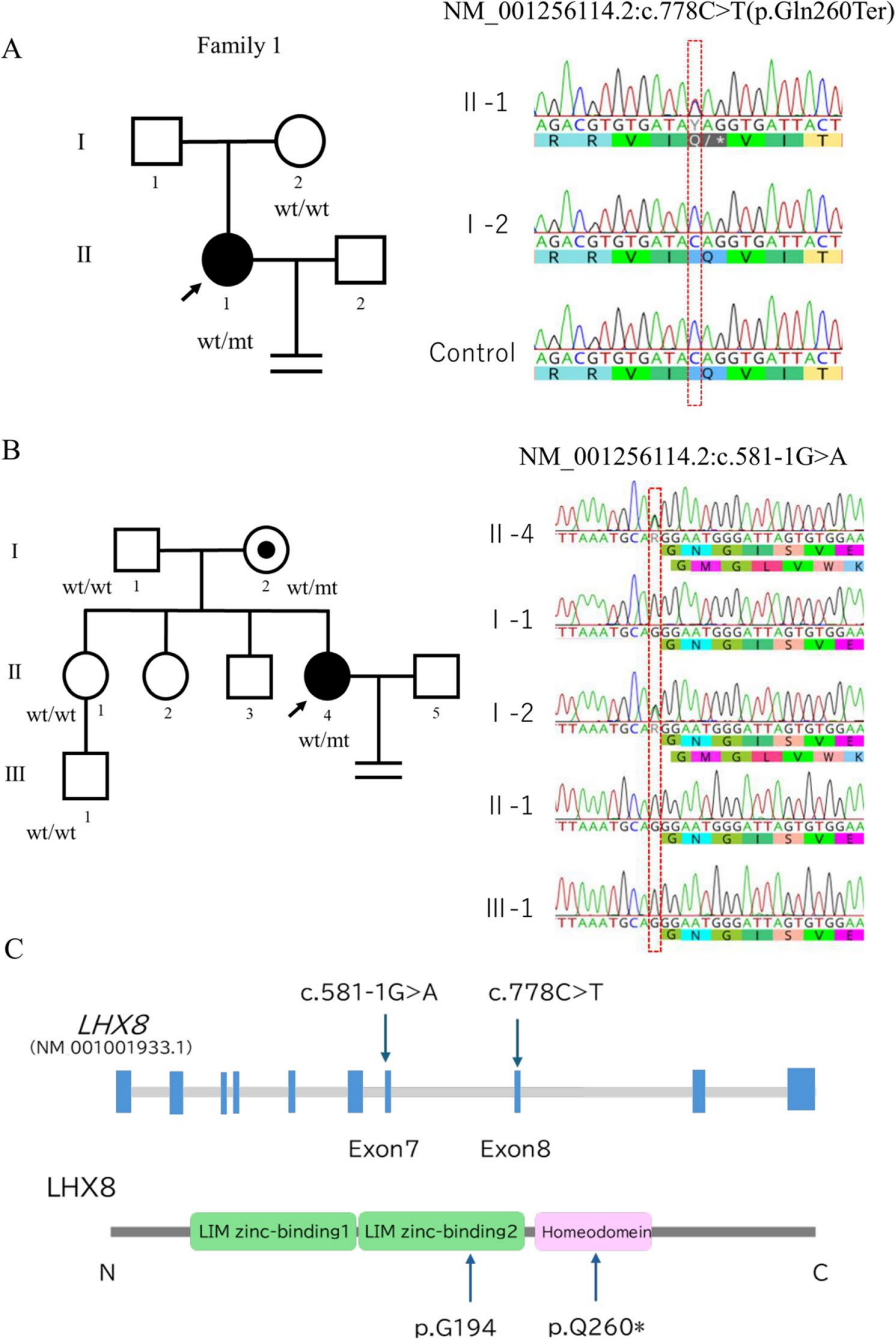
Identification of *LHX8* variants in two families. **A**. Pedigree of family 1 and Sanger sequencing chromatograms showing the heterozygous LHX8 nonsense variant c.778 C > T (p.Gln260Ter) in the female proband (II-1). The variant was not detected in the proband’s mother (I-2). **B**. Pedigree of family 2 and Sanger sequencing chromatograms showing the heterozygous LHX8 splice site variant c.581-1G > A in the female proband (II-4) and her mother (I-2). This variant was not detected in the father (I-1), the proband’s sister (II-1), or her nephew (III-1), as indicated by wild-type sequences identical to the control. **C**. Schematic representation of the LHX8 gene structure and protein domains showing the location of the identified variants. Arrows indicate the positions of the variants

Two patients with primary infertility from different families were recruited, each from a different fertility clinic. In Family 1, peripheral blood samples were collected from the proband (II-1) and her mother (I-2) (Fig. 1A). In Family 2, samples were obtained from the proband (II-4), her mother (I-2), sister (II-1), and nephew (III-1) (Fig. 1B). Comprehensive pedigree information was documented, including fertility history, age at conception, and any history of reproductive disorders. Genomic DNA was extracted from all samples for genetic testing and a segregation analysis. All participants provided written informed consent, and this study was approved by the Institutional Review Board of Fujita Health University (HG24-014).

### Clinical protocol and assisted reproductive procedures

A controlled ovarian stimulation was started on day 3 of the menstrual cycle using one of three protocols: the gonadotropin-releasing hormone (GnRH) agonist protocol, GnRH antagonist protocol, or progestin-primed ovarian stimulation (PPOS). Protocol selection was based on each subject’s characteristics, including age, anti-Müllerian hormone (AMH) levels, and previous ovarian responses. In the GnRH agonist protocol, patients received buserelin acetate (Suprecur; Mochida Pharmaceutical, Tokyo, Japan) daily starting from day 3 of the menstrual cycle and continuing throughout the stimulation period. In the GnRH antagonist protocol, cetrorelix acetate (Cetrotide; Merck Biopharma, Tokyo, Japan) was administered when the leading follicle reached 14 mm in diameter. In PPOS, dydrogesterone (Duphaston; Mylan EPD, Tokyo, Japan) was administered from cycle day 3 until the trigger day. Follicular development was monitored by transvaginal ultrasonography and serum estradiol measurements. When the two leading follicles reached at least 20 mm in diameter, human chorionic gonadotropin (hCG; Mochida Pharmaceutical, Tokyo, Japan) 5000 IU was administered. Oocyte retrieval was performed 36–38 h after the hCG trigger under transvaginal ultrasound guidance with sedation. Retrieved oocytes were inseminated by conventional in vitro fertilization (IVF) or intracytoplasmic sperm injection based on semen parameters or fertilization rates. The fertilization status was assessed 16–18 h post-insemination by checking for the presence of two pronuclei.

### Whole-exome sequencing (WES) and variant analysis

Genomic DNA was extracted from 400 µL of peripheral blood or saliva samples using the magLEAD system (Precision System Science, Chiba, Japan). WES was performed on the NovaSeq 6000 platform (Illumina, San Diego, CA). Raw sequnce data (FASTQ files) were aligned to the human reference genome (UCSC hg38/GRCh38). A coverage analysis revealed that at least 97% of exons had > 30× coverage. Variant calling and filtering were conducted based on quality metrics, minor allele frequency (MAF), and the predicted functional impact. Allele frequencies were obtained from gnomAD v4.1.0 (807,162 exomes and 76,215 genomes from diverse ancestries; https://gnomad.broadinstitute.org/), and from the Tohoku Medical Megabank Organization database, ToMMo 8.3KJPNv2 (whole-genome data from 8,380 Japanese individuals; https://jmorp.megabank.tohoku.ac.jp/help/tutorial). These databases include male and female participants across various age groups, though reproductive phenotype data are not systematically recorded. An MAF cut-off of < 0.01 was applied to both databases.

Candidate variants identified by WES were validated by Sanger sequencing. PCR primers flanking the regions of interest were designed using Primer3 software (https://bioinfo.ut.ee/primer3/). After amplification, PCR products were purified and sequenced using the SeqStudio Genetic Analyzer (Thermo Fisher Scientific, Waltham, MA). The pathogenicity of validated variants was classified according to the 2015 ACMG/AMP guidelines (American College of Medical Genetics and Genomics / Association for Molecular Pathology). Computational tools, such as gnomAD metrics (probability of being loss-of-function intolerant (pLI) and loss-of-function observed/expected upper bound fraction (LOEUF) scores; https://gnomad.broadinstitute.org/), DECIPHER prediction tools (probability of haploinsufficiency (pHaplo), probability of triplosensitivity, and probability of loss-of-function mechanism (pLOF) scores) https://www.deciphergenomics.org/), and SpliceAI (https://spliceailookup.broadinstitute.org/), were used to predict the functional impact of the variants identified.

### Minigene analysis

A minigene splicing assay was performed as previously described [9]. Briefly, the *LHX8* genomic region encompassing exon 6, intron 6, and exon 7 with flanking sequences was amplified from patient and control genomic DNA using primers containing XhoI and SpeI restriction sites. The resulting PCR products were subcloned into the multiple cloning site of the pET01 Exontrap vector (MoBiTec). These plasmids were transfected into HEK293 cells using Lipofectamine 3000 (Thermo Fisher Scientific). Cells were harvested 48 h after transfection, and total RNA was isolated using the RNeasy Mini Kit (Qiagen). First-strand cDNA was synthesized using SuperScript III (Invitrogen) with oligo(dT) primers. RT-PCR was performed with vector-specific primers, and PCR products were analyzed by 2% agarose gel electrophoresis and direct Sanger sequencing to evaluate splicing patterns.

## Results

### Clinical phenotypes

The clinical characteristics of the study patients are summarized in Table 1. In Family 1, the patient (II-1) was a 31-year-old female who presented with a 5-year history of infertility. Physical examination and routine laboratory tests, including thyroid function and hormone profiles, were unremarkable. The patient had no history of systemic diseases, autoimmune disorders, or other reproductive tract abnormalities. She had been diagnosed with unexplained infertility and had undergone three months of timed intercourse followed by 12 cycles of intrauterine insemination; however, pregnancy was not achieved. Consequently, the decision was made to proceed with ART. The patient had a normal ovarian reserve with an AMH level of 2.02 ng/mL. All parameters (concentration, motility, and morphology) were within normal ranges in her spouse’s semen.

**Table 1:** Clinical characteristics of study patients

	Age (years)	AMH (ng/mL)	Duration of infertility (years)	Number of retrievals	Total number of oocytes	GV oocyte (*n*)	MI oocyte (*n*)	MII oocyte (*n*)	Oocytes with an abnormal morphology (*n*)	Fertilized oocytes (*n*)	Viable embryos (*n*)	Features
Family 1 (Ⅱ-1)	31	2.02	5	6	126	15	29	54	21	51	1	Frequent cytoplasmic vacuoles and polyspermy in PVS
Family 2 (Ⅱ-4)	27	1.62	4	7	33	2	0	15	14	5	0	Mainly polyspermy in PVS

*Abbreviations*: *GV* Germinal Vesicle, *MI* Metaphase I, *MII* Metaphase II, *PVS* perivitelline space

**Fig. 2:**
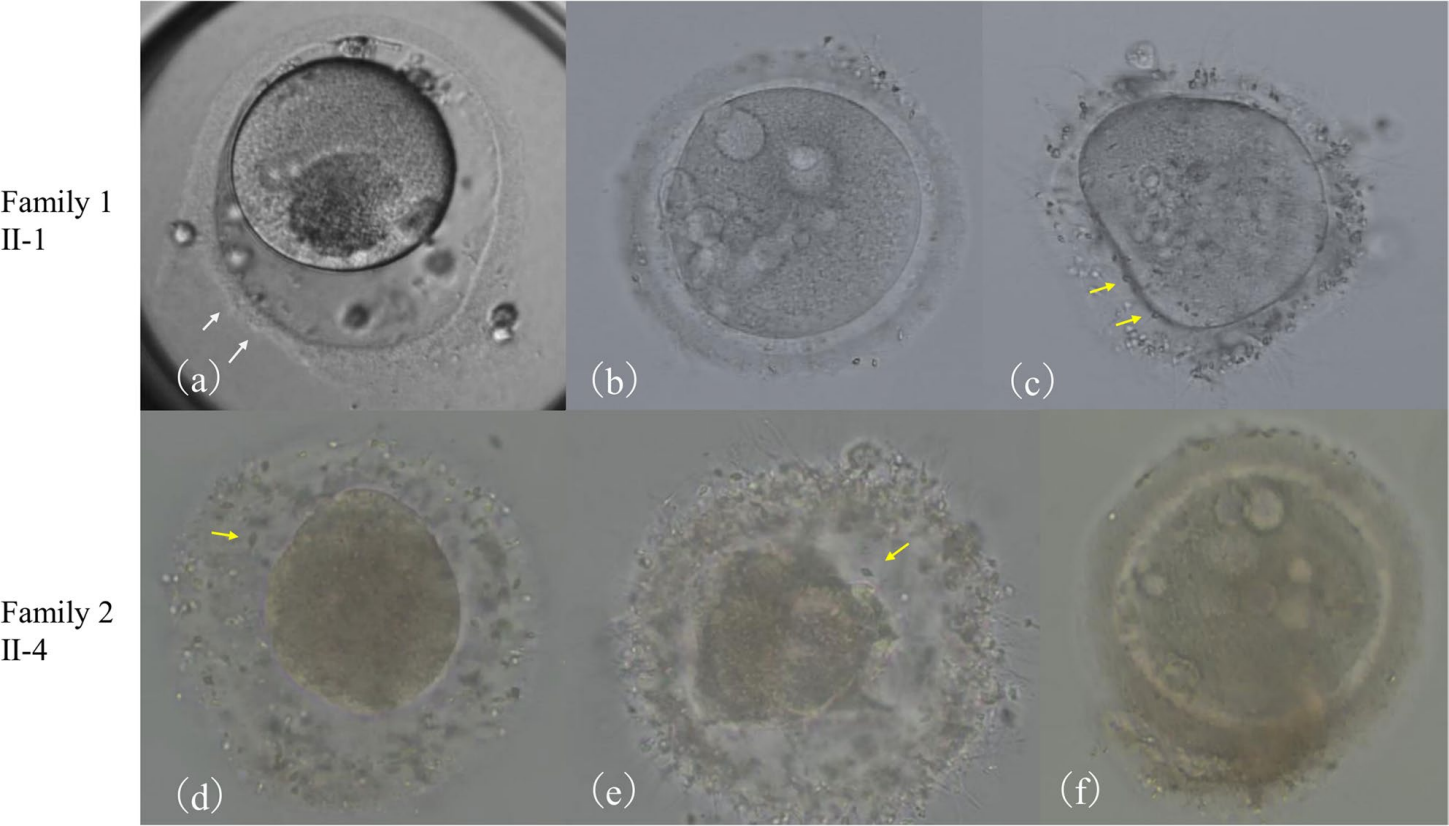
Morphological abnormalities in proband oocytes during fertilization. Representative images of oocytes collected from family 1 proband (II-1, a-c) and family 2 proband (II-4, d-f). **a** Oocyte with abnormal zona pellucida with partial thinning and degenerative changes (white arrows). **b** Oocyte from family 1 proband with multiple cytoplasmic vacuoles. **c** Oocyte with multiple spermatozoa in the perivitelline space (yellow arrows). **d**, **e** Oocytes from family 2 proband with multiple spermatozoa in the perivitelline space (yellow arrows). **f** Oocyte from family 2 proband with multiple cytoplasmic vacuoles

Six oocyte retrieval cycles were performed, during which 126 oocytes were retrieved (15 GV, 29 MI, and 54 metaphase II (MII) oocytes). Among these oocytes, 21 (16.7%) had an abnormal morphology characterized by an abnormal zona pellucida with partial thinning (Fig. 2a), multiple cytoplasmic vacuoles (Fig. 2b), and multiple spermatozoa penetration into the perivitelline space after conventional IVF (Fig. 2c). In fertilized oocytes, cytoplasmic vacuoles increased during the pronuclear stage. Despite multiple attempts, with 51 oocytes being successfully fertilized, only one viable embryo developed to a day 6 blastocyst with Gardner grade 3BC. This blastocyst was transferred, but did not result in pregnancy. 

In Family 2, the patient (II-4) was a 27-year-old female with a 4-year history of infertility. Physical examination and routine laboratory tests, including thyroid function and hormone profiles, were unremarkable. She had no history of systemic diseases, autoimmune disorders, or other reproductive tract abnormalities, and her AMH level was 1.62 ng/mL. The spousal semen analysis revealed that all parameters were within normal ranges. Thirty-three oocytes were retrieved (2 GV, 0 MI, and 15 MII oocytes) from seven cycles. Similar to Family 1, many oocytes (14/33, 42.4%) had an abnormal morphology. These abnormalities were characterized predominantly by multiple sperm penetration into the perivitelline space following conventional IVF (Fig. 2d, e) and cytoplasmic vacuoles at the pronuclear stage (Fig. 2f). Five oocytes achieved fertilization; however, no viable embryos were obtained.

The patient’s mother (Family2, I-2) with the same variant had given birth to four children at the ages of 22, 24, 30, and 32 years. She had no history of infertility treatment and experienced menopause at approximately 50 years of age. The patient’s sister (II-1) had conceived naturally at the age of 28 years without any infertility issues. Her other siblings (II-2 and II-3) were unmarried at the time of the analysis.

### Variant analysis

A genetic analysis identified heterozygous *LHX8* variants in both probands (Table 2). In Family 1, the proband (II-1) carried the nonsense variant c.778 C > T, which was not found in her mother (I-2). Since a paternal sample was unavailable, this variant indicated *de novo* or paternal inheritance (Fig. 1A). In Family 2, the proband (II-4) carried the heterozygous splice site variant c.581-1G > A, which was inherited from her mother (I-2). Sanger sequencing confirmed that the proband’s sister (II-1) and her son (III-1) were both negative for this variant (Fig. 1B).

**Table 2:** Overview of *LHX8* variants

	Variants	Variant type	Zygosity	gnomAD	ToMMo	ACMG criteria	ACMG pathogenicity class
Family 1 (Ⅱ-1)	c.778 C > T(p.Q260*)	Nonsense	Heterozygous	NA	NA	PVS1, PM2	Likely Pathogenic
Family 2 (Ⅱ-4)	c.581-1G > A	Splicing	Heterozygous	NA	NA	PVS1, PM2	Likely Pathogenic

*NA* not available

The nonsense variant c.778 C > T (p.Gln260Ter) in Family 1 introduced a premature termination codon in exon 7. Regarding the splice site variant c.581-1G > A in Family 2, a sequence analysis confirmed that it affected the invariant G nucleotide at the − 1 position of the canonical splice acceptor site. This nucleotide position showed complete conservation across vertebrate species in the *LHX8* gene. Both variants were absent from population databases (gnomAD and ToMMo), indicating that they are not common polymorphisms in the general population.

To examine the pathogenicity of the *LHX8* variants in the present study, we utilized complementary metrics from multiple databases. *LHX8* demonstrated an intolerance to loss-of-function variants, with gnomAD pLI of 0.97 and LOEUF of 0.52. A DECIPHER analysis indicated pHaplo of 0.75 and pLOF of 0.655.

Regarding the splice site variant c.581-1G > A, SpliceAI predicted high probabilities of acceptor loss (0.99) and splice loss (0.85). Additionally, this variant showed a significant acceptor gain score (0.94) 2 bp from the original site, suggesting the activation of a cryptic splice site.

According to the ACMG/AMP guidelines, both variants were classified as likely pathogenic, fulfilling criteria PVS1 and PM2 in both cases (Table 2).

### Functional analysis of the c.581-1G > A splice site variant

**Fig. 3:**
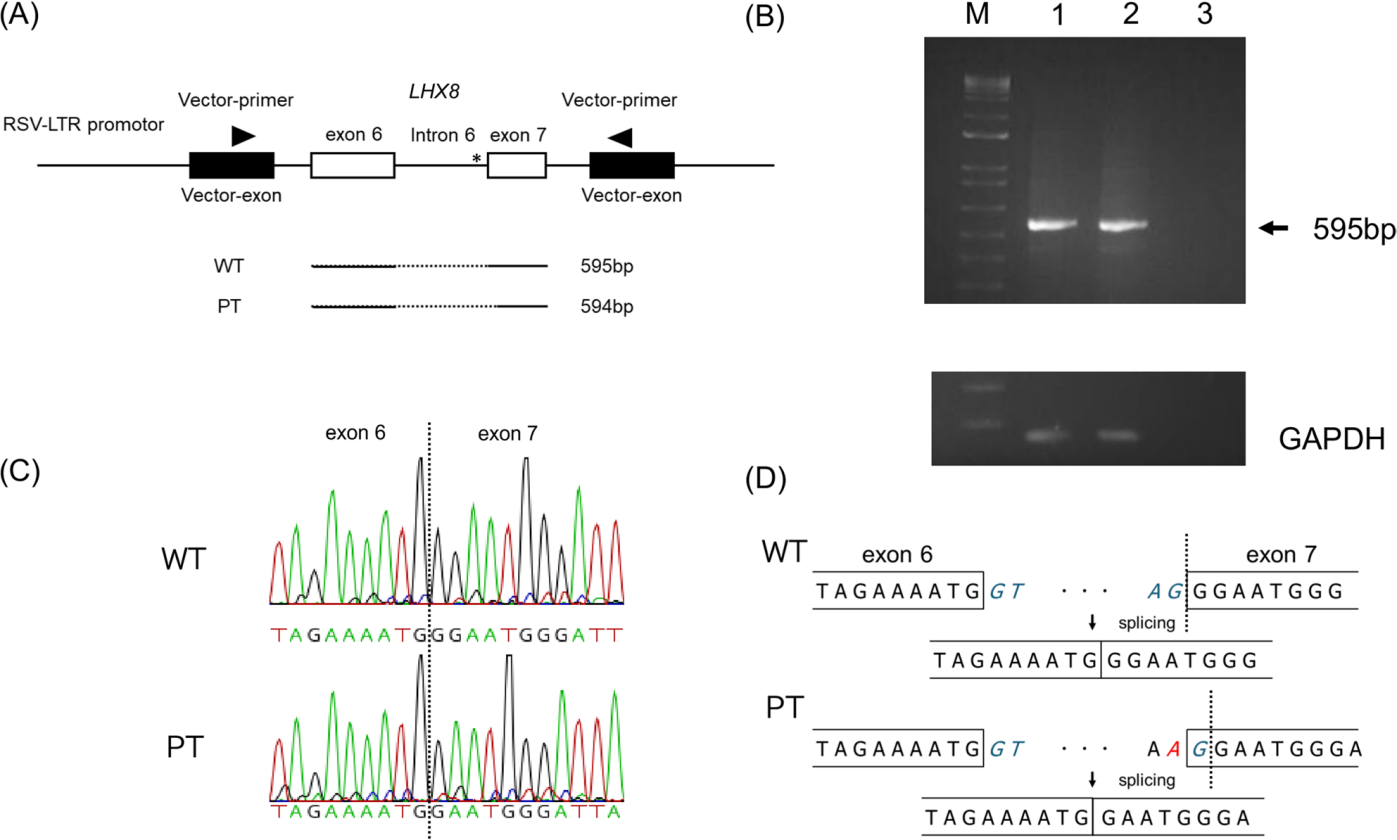
Functional analysis of the *LHX8* c.581-1G > A splice site variant using minigene assays. **A** Schematic representation of the minigene construct and RT-PCR analysis. The LHX8 genomic fragment containing exons 6 and 7 with intervening intron 6 was cloned into the pET01 Exontrap vector. Expected PCR product sizes are shown for the WT (595 bp) and PT (594 bp) constructs. Dotted lines indicate splicing events; solid lines indicate retained sequences. *Asterisk indicates the variant. **B** 2% agarose gel electrophoresis of RT-PCR products from HEK293 cells transfected with WT and PT constructs. Lane M, molecular weight marker; lane 1, WT construct; lane 2, PT construct; lane 3, mock transfection control (NC). GAPDH was used as a loading control. **C** Sanger sequencing chromatograms showing the splice junction region. Upper panel: WT sequence showing normal splicing between exons 6 and 7. Lower panel: PT sequence showing aberrant splicing. **D** Schematic illustration of the splicing pattern. The c.581-1G > A variant abolishes the canonical splice acceptor site, resulting in the activation of a cryptic splice site located 2 bp downstream. This results in a 1-bp deletion in the mature transcript from the WT sequence. Dotted lines indicate the splicing events

To investigate the functional consequences of the c.581-1G > A variant on *LHX8* splicing, we performed minigene splicing assays using the Exontrap system. The genomic fragment containing *LHX8* exons 6 and 7 with the intervening intron 6 was cloned into the pET01 vector and transfected into HEK293 cells. The RT-PCR analysis of transfected cells revealed distinct splicing patterns between the wild-type (WT) and patient (PT) constructs. The WT minigene produced the expected transcript containing exons 6 and 7, with the normal splicing of intron 6 (Fig. 3A, lane 2). No clear differences were detected in PCR product sizes between WT and PT constructs by agarose gel electrophoresis (Fig. 3B). Sanger sequencing of RT-PCR products revealed that the c.581-1G > A variant resulted in the use of an aberrant splice site located 1 bp downstream of the original acceptor site (Fig. 3C-D). This led to a 1-bp deletion in the mature transcript from the WT sequence. This splicing pattern was consistently observed across three independent experiments and aligned with SpliceAI predictions.

## Discussion

We identified two women with primary infertility who carry novel *LHX8* variants, each with high percentages of degenerated and immature oocytes. Despite different variants, the cases showed two common features: accumulation of sperm within the perivitelline space and increased cytoplasmic vacuoles in oocytes at the pronuclear stage.

These morphological abnormalities may be understood in the context of the function of *LHX8*. *LHX8* acts as a key transcription factor in early oogenesis and interacts with *SOHLH1* to form a nuclear complex [7, 10]. This complex coordinates the expression of downstream genes that are essential for oocyte growth and differentiation, including *Zp1* and *Zp3* [11]. Zona pellucida proteins form the glycoprotein matrix around the oocyte and have central roles in species specific sperm recognition, binding, and prevention of polyspermy [12]. The observed sperm accumulation suggests zona pellucida dysfunction, which may reflect altered expression or function of these proteins. Cytoplasmic vacuoles are established markers of reduced oocyte competence and are associated with lower fertilization rates and poorer outcomes [13]. Collectively, these abnormalities support the view that *LHX8* regulates oocyte quality through multiple pathways. Further functional studies are needed to clarify the diverse effects of *LHX8* variants in oogenesis.

Our results extend previous reports on *LHX8* related infertility. Zhao et al. described heterozygous loss of function *LHX8* variants, including splicing, nonsense, and frameshift variants, caused human infertility [8]. The type of variant may modify the phenotype, as missense variants have been reported in patients with POI [14], and common polymorphisms have been associated with the risk of POI in genome-wide studies [15]. In our study, both patients carried null variants and had normal ovarian reserve, with infertility as the only clinical finding.

The minigene assay confirmed the use of a cryptic splice acceptor site 2 bp downstream, resulting in a 1 bp deletion and frameshift as predicted by SpliceAI. Despite this functional evidence, incomplete penetrance was evident in family 2, where the mother carried the same variant and was fertile. The pedigree, in which the proband’s sister and her child did not inherit the variant and had normal fertility, supports incomplete penetrance. This has practical implications for counseling because clinical expression can be modified by trans-acting factors [16, 17], epigenetic changes, and environmental factors such as age and hormonal prolife.

These observations also support interspecies differences in dosage sensitivity. While homozygous deletion of *Lhx8* results in a phenotype in mice, heterozygous *LHX8* variants appear to be sufficient to cause reproductive abnormalities in humans [8, 18]. This pattern, together with high constraint metrics such as a pLI of 0.97 and a pHaplo of 0.75 in our cases, suggests that human oogenesis is sensitive to *LHX8* dosage. At the cellular level, recent work in mice links Lhx8 deficiency to altered autophagy [19]. The vacuoles we observed may reflect a related disruption in cellular quality control.

Clinically, our data suggest that *LHX8* testing may be considered for women with recurrent OMA, particularly when cytoplasmic vacuoles or abnormal perivitelline sperm accumulation are present. However, this study has several limitations that should be considered. First, the paternal sample was not available in Family 1, preventing definitive determination of the mode of inheritance. Second, phenotypically confirmed fertile controls were unavailable, and we therefore used population databases to assess variant frequency. The complete absence of these variants in over 800,000 individuals supports their pathogenicity.

## Conclusion

We report two clinical cases with novel *LHX8* variants (c.778 C > T and c.581-1G > A) that are likely pathogenic according to ACMG and AMP guidelines. The consistent morphological and developmental findings support the central role of *LHX8* in oocyte maturation. These features may guide genetic testing and counseling for unexplained infertility.

## Supplementary Information

Supplementary Material 1: Supplementary Figure S1. Full uncropped agarose gel images corresponding to Fig. 3B, including all lanes and DNA size markers.

## Data Availability

The datasets generated and/or analyzed during the present study are available from the corresponding author upon reasonable request.

## References

[1] Liang Y, Huang J, Zhao Q, Mo H, Su Z, Feng S, et al. Global, regional, and National prevalence and trends of infertility among individuals of reproductive age (15–49 years) from 1990 to 2021, with projections to 2040. Hum Reprod. 2025;40:529–44. 39752330 10.1093/humrep/deae292

[2] Hatırnaz Ş, Hatırnaz ES, Ellibeş Kaya A, Hatırnaz K, Soyer Çalışkan C, Sezer Ö, et al. Oocyte maturation abnormalities - A systematic review of the evidence and mechanisms in a rare but difficult to manage fertility pheneomina. Turk J Obstet Gynecol. 2022;19:60–80. 35343221 10.4274/tjod.galenos.2022.76329 PMC8966321

[3] Van Der Kelen A, Uyttebroeck S, Van de Voorde S, Picchetta L, Segers I, Vlaeminck J, et al. Oocyte/zygote/embryo maturation arrest: a clinical study expanding the phenotype of NOBOX variants. J Assist Reprod Genet. 2025;42:763–71. 39871066 10.1007/s10815-025-03402-y PMC11950555

[4] Feng R, Sang Q, Kuang Y, Sun X, Yan Z, Zhang S, et al. Mutations in TUBB8 and human oocyte meiotic arrest. N Engl J Med. 2016;374:223–32. 26789871 10.1056/NEJMoa1510791 PMC4767273

[5] Kitanaka J, Takemura M, Matsumoto K, Mori T, Wanaka A. Structure and chromosomal localization of a murine LIM/homeobox gene, Lhx8. Genomics. 1998;49:307–9. 9598319 10.1006/geno.1998.5203

[6] Fu L, Zhang M, Mastrantoni K, Perfetto M, Wei S, Yao J. Bovine Lhx8, a germ cell-specific nuclear factor, interacts with Figla. PLoS ONE. 2016;11:e0164671. 27716808 10.1371/journal.pone.0164671 PMC5055334

[7] Choi Y, Ballow DJ, Xin Y, Rajkovic A. Lim homeobox gene, lhx8, is essential for mouse oocyte differentiation and survival. Biol Reprod. 2008;79:442–9. 18509161 10.1095/biolreprod.108.069393 PMC2710541

[8] Zhao L, Li Q, Kuang Y, Xu P, Sun X, Meng Q, et al. Heterozygous loss-of-function variants in LHX8 cause female infertility characterized by oocyte maturation arrest. Genet Med. 2022;24:2274–84. 36029299 10.1016/j.gim.2022.07.027

[9] Bolor H, Mori T, Nishiyama S, Ito Y, Hosoba E, Inagaki H, et al. Mutations of the SYCP3 gene in women with recurrent pregnancy loss. Am J Hum Genet. 2009;84:14–20. 19110213 10.1016/j.ajhg.2008.12.002 PMC2668043

[10] Wang Z, Liu C-Y, Zhao Y, Dean J, FIGLA. LHX8 and SOHLH1 transcription factor networks regulate mouse oocyte growth and differentiation. Nucleic Acids Res. 2020;48:3525–41. 32086523 10.1093/nar/gkaa101 PMC7144910

[11] Pangas SA, Choi Y, Ballow DJ, Zhao Y, Westphal H, Matzuk MM, et al. Oogenesis requires germ cell-specific transcriptional regulators Sohlh1 and Lhx8. Proc Natl Acad Sci U S A. 2006;103:8090–5. 16690745 10.1073/pnas.0601083103 PMC1472434

[12] Wassarman PM. Zona pellucida glycoproteins. J Biol Chem. 2008;283:24285–9. 18539589 10.1074/jbc.R800027200 PMC2528931

[13] Nikiforov D, Grøndahl ML, Hreinsson J, Andersen CY. Human oocyte morphology and outcomes of infertility treatment: A systematic review. Reprod Sci. 2022;29:2768–85. 34816375 10.1007/s43032-021-00723-y

[14] Bouilly J, Beau I, Barraud S, Bernard V, Azibi K, Fagart J, et al. Identification of multiple gene mutations accounts for a new genetic architecture of primary ovarian insufficiency. J Clin Endocrinol Metab. 2016;101:4541–50. 27603904 10.1210/jc.2016-2152

[15] Qin Y, Zhao H, Kovanci E, Simpson JL, Chen Z-J, Rajkovic A. Analysis of LHX8 mutation in premature ovarian failure. Fertil Steril. 2008;89:1012–4. 17624344 10.1016/j.fertnstert.2007.04.017 PMC2680741

[16] Cooper DN, Krawczak M, Polychronakos C, Tyler-Smith C, Kehrer-Sawatzki H. Where genotype is not predictive of phenotype: towards an Understanding of the molecular basis of reduced penetrance in human inherited disease. Hum Genet. 2013;132:1077–130. 23820649 10.1007/s00439-013-1331-2 PMC3778950

[17] Castel SE, Cervera A, Mohammadi P, Aguet F, Reverter F, Wolman A, et al. Modified penetrance of coding variants by cis-regulatory variation contributes to disease risk. Nat Genet. 2018;50:1327–34. 30127527 10.1038/s41588-018-0192-y PMC6119105

[18] Ren Y, Suzuki H, Jagarlamudi K, Golnoski K, McGuire M, Lopes R, et al. Lhx8 regulates primordial follicle activation and postnatal folliculogenesis. BMC Biol. 2015;13:39. 26076587 10.1186/s12915-015-0151-3 PMC4487509

[19] D’Ignazio L, Michel M, Beyer M, Thompson K, Forabosco A, Schlessinger D, et al. Lhx8 ablation leads to massive autophagy of mouse oocytes associated with DNA damage. Biol Reprod. 2018;98:532–42. 29329412 10.1093/biolre/iox184 PMC6279113

