# Supplementary figures and images for: Novel LHX8 variants associated with distinctive oocyte morphological abnormalities and maturation arrest in primary infertility
Source: J Ovarian Res. 2026 Jan 21;19:61. doi: 10.1186/s13048-026-01978-2 (PMC12911217; doi:10.1186/s13048-026-01978-2)

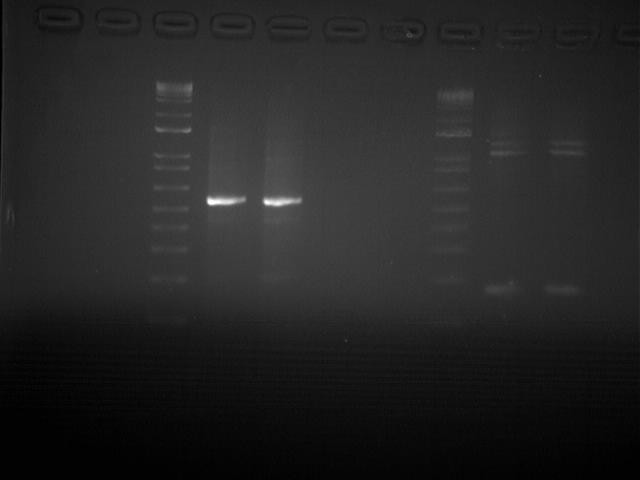

Supplement: Supplementary file 1 — Supplementary Material 1: Supplementary Figure S1. Full uncropped agarose gel images corresponding to Fig. 3B, including all lanes and DNA size markers. [file 13048_2026_1978_MOESM1_ESM.tif]
